# The 50 most cited articles on patellofemoral instability: A bibliometric and network analysis of research trends and impact

**DOI:** 10.1016/j.jor.2025.06.026

**Published:** 2025-07-01

**Authors:** Alexander Price, Nicolaas Kotze, Xander van Heerden, Gerard A. Sheridan

**Affiliations:** a Department of Trauma & Orthopaedics, University Hospital Galway, Galway, Ireland; b Royal College of Surgeons Ireland, Dublin, Ireland; c University of Galway, Galway, Ireland

**Keywords:** Patellofemoral instability, Bibliometric analysis, MPFL reconstruction, Citation trends, Conservative treatment

## Abstract

**Background:**

Patellofemoral instability remains a complex clinical challenge, especially among adolescents and young adults. While research in this area has expanded considerably, few studies have comprehensively examined the most influential literature shaping current knowledge and guiding clinical practice.

**Methods:**

The top 50 most cited articles related to patellofemoral instability were identified using Publish or Perish software (Version 8, Harzing.com) to access data from Google Scholar. Citation metrics, study characteristics (e.g. design, level of evidence, origin, journal), and thematic foci were extracted. Keyword network mapping was conducted using Gephi to identify dominant research themes.

**Results:**

Citation counts ranged from 179 to 2,295, with a median of 276. Most studies were published after 2005 and were predominantly Level IV and V evidence. Surgical reconstruction, especially medial patellofemoral ligament (MPFL) procedures, was the prevailing research focus. Geographically, 40 % of studies originated from the United States of America (USA), followed by France and the United Kingdom (UK). Thematic clustering revealed three main domains: surgical reconstruction, anatomical diagnostics, and clinical outcomes. Non-operative treatment modalities and rehabilitation were notably under investigated.

**Conclusion:**

This study provides an up-to-date and comprehensive analysis of the most influential literature regarding patellofemoral instability. It highlights persistent gaps in high-level evidence, conservative treatment approaches, and global representation. These findings offer a strategic direction for future research, emphasizing the need for high-level evidence that is both inclusive and patient-focused to better guide clinical practice.

## 1 Introduction

Patellofemoral instability is a multifactorial and complex orthopaedic condition predominantly affecting adolescents and young adults, especially those engaged in sports and other physical activities. ^1^ It most commonly follows a traumatic lateral patellar dislocation and is characterized by recurrent instability episodes, anterior knee pain, functional limitations, and reduced quality of life. ^2^^,^^3^ This condition poses significant challenges for clinicians, as patients often experience persistent symptoms that impact their daily activities and athletic performance. ^4^ Consequently, patellofemoral instability represents a frequent cause for referral to orthopaedic and sports medicine specialists, with long-term implications for joint health and physical function. Risk factors such as trochlear dysplasia, patella alta, lateralized tibial tubercle, increased Q-angle, and generalized ligamentous laxity have been identified as critical contributors to abnormal patellar tracking and containment failure during early knee flexion. ^5^^,^^6^ Clinical diagnosis is supported by physical examination and imaging studies, with measurements like the tibial tubercle–trochlear groove (TT-TG) distance and sulcus angle being widely used. However, there remains considerable debate about the optimal thresholds and their predictive value, reflecting the heterogeneity of the condition. ^7^^,^^8^ Over the past twenty years there has been a marked increase in studies focused on patellofemoral instability. Advances in imaging modalities, particularly magnetic resonance imaging (MRI), have improved the sensitivity of anatomical abnormalities and soft tissue injuries associated with instability. ^9^ Simultaneously, the development and validation of patient-reported outcome measures (PROMs), such as the Kujala score and the Banff Patellofemoral Instability Instrument (BPII), have provided tools to better quantify symptom severity and treatment outcomes. ^10^ MPFL reconstruction has emerged as the principal surgical intervention for recurrent patellofemoral instability, aiming to restore the native soft tissue constraints and prevent lateral patellar displacement during knee flexion. ^11^ As a result, a large body of literature has explored aspects of MPFL reconstruction, including graft choice, anatomical tunnel positioning, fixation techniques, and postoperative outcomes. ^12^^,^^13^ Despite the surgical focus, conservative treatment modalities such as physical therapy, neuromuscular training, bracing, and taping remain relevant, especially for patients with first-time dislocations. However, these non-operative approaches are underrepresented in high-impact research publications. ^14^^,^^15^ The overall quality of evidence in the field is heterogeneous. Many of the highly cited studies are retrospective, with diverse patient populations and variable follow-up durations. ^16^ Randomized controlled trials (RCT's) are scarce, limiting the strength of evidence guiding best practices and contributing to variability in treatment approaches. Bibliometric analysis provides an objective approach to evaluate scholarly influence and research trends by measuring citation patterns, author contributions, institutional output, and the development of key topics. ^17^^,^^18^ Recent advances using advanced computational analysis of research abstracts to identify commonly linked medical terms and research topics have enhanced these methods, allowing for more detailed identification of research clusters and emerging areas of focus. Although bibliometric studies have been widely applied to orthopaedic subspecialties such as anterior cruciate ligament reconstruction, rotator cuff surgery, and total joint arthroplasty, focused bibliometric evaluations of patellofemoral instability are only now beginning to emerge. 19, 20, 21

The present study utilizes a comprehensive dataset gathered through Publish or Perish software accessing Google Scholar, including articles published up to May 2025. This broader and more inclusive data source captures a wider spectrum of influential publications, including those absent on traditional databases. We utilize advanced computational analysis of keywords and their co-occurrence patterns to identify key thematic areas within the literature. Lastly, we conducted a detailed evaluation of study designs and levels of evidence to better interpret the quality of the most impactful research. By consolidating citation metrics, authorship and geographic patterns, thematic clusters, and methodological insights, this bibliometric analysis provides a comprehensive and current overview of patellofemoral instability research. Our findings aim to inform clinicians and researchers, guiding future investigations toward a more inclusive patient population, incorporating rigorous methodological standards to provide an evidence-based approach to the management of this challenging condition.

## 2 Methods

### 2.1 Study selection and search strategy

A comprehensive literature search was conducted in May 2025 using Publish or Perish software (Version 8, Harzing.com) to query Google Scholar. The search strategy applied Boolean operators, simple connectors such as “and,” “or,” and “not”, to combine or exclude keywords and improve search precision. Key terms related to patellofemoral instability were used, including “patellofemoral instability,” “patellar dislocation,” “medial patellofemoral ligament reconstruction,” “MPFL.” No restrictions were placed on publication date or journal source to ensure a broad and inclusive representation of relevant literature.

Exact search string used: “Patellofemoral instability” OR “Patellar instability” OR “Patellar dislocation” OR “Medial Patellofemoral Ligament” OR “MPFL reconstruction”

Articles were ranked in descending order based on total citation count. To ensure methodological precision, two independent reviewers screened the titles and abstracts for relevance using predefined inclusion and exclusion criteria. This process was conducted systematically to minimize selection bias. Discrepancies, if any, were resolved through consensus, and the senior author was consulted if disagreement persisted.

### 2.2 Eligibility criteria

To ensure consistency, relevance, and scientific quality, studies were included if they:

• Were original research articles with a primary focus on patellofemoral instability, covering areas such as diagnosis, treatment strategies, biomechanics, or clinical outcomes.
• Were published in peer-reviewed academic journals.
• Were written in English.
• Had full-text availability to allow comprehensive methodological and thematic review.

Studies were excluded if they:

• Were editorials, opinion pieces, or conference abstracts, as these do not contain original primary data.
• Were animal or in vitro studies that did not directly address clinical patellofemoral instability.
• Lacked accessible full-text versions, which would impede critical assessment and data collection.

The **top 50 most cited articles** were selected for inclusion in the final bibliometric and thematic analysis. **All selected articles met the predefined eligibility criteria** and no articles were excluded due to duplication or irrelevance, demonstrating the specificity and robustness of the search strategy.

### 2.3 Data extraction and study characteristics

For each included publication, a standardized data extraction form was used to collect key study characteristics:

• Article title, full list of authors, and year of publication.
• Publishing journal and institutional affiliation of the first author.
• Country of origin based on first author affiliation.
• Study design (e.g. RCT's, prospective or retrospective cohort, case series).
• Level of evidence, assigned independently by two reviewers based on the Oxford Centre for Evidence-Based Medicine (OCEBM) guidelines. ^22^
• Citation metrics, including total citation count and citation density (citations per year). • Primary research focus (e.g. surgical techniques, imaging, complications, or rehabilitation).
• Sample size, demographics, and follow-up duration, where available.

### 2.4 Statistical and network analysis

Descriptive statistics, including medians, frequencies, and interquartile ranges, were calculated using **StataSE Version 18.0** (StataCorp LLC, College Station, TX, USA) to summarize citation metrics, study designs, and geographic contributions. **Microsoft Excel (Version 16.97, Microsoft Corporation, Redmond, WA, USA)** was used to generate statistical graphical visualizations.

To explore relationships between study themes, **keyword co-occurrence analysis** was conducted by examining the abstract content of each article. **Gephi software (Version 0.10.1, Gephi Consortium)** was used to construct visual networks where nodes indicated frequently occurring keywords, and lines represented co-occurrence between terms. Clustering methods identified major thematic areas.

### 2.5 Ethical considerations

This study relied exclusively on publicly accessible bibliometric data and did not involve human participants or personal health information. As such, it was exempt from institutional review board approval and adhered to ethical standards governing secondary research.

## 3 Results

### 3.1 Citation metrics and publication trends

The top 50 most cited articles related to patellofemoral instability were published between 1994 and 2022. The total number of citations ranged from 179 to 2295 (Median: 276; IQR 204–417). The most cited article, authored by Dejour et al., in 1994, introduced a radiographic classification of trochlear dysplasia and remains a cornerstone in the diagnosis of patellar instability. ^3^

**Fig. 1:**
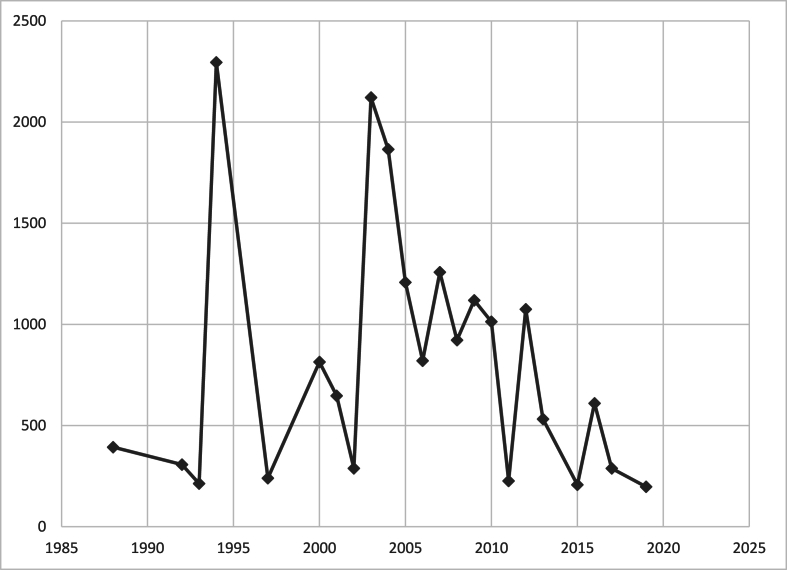
Total citations by year.

Most articles (68 %) were published after 2005, reflecting a growing academic interest in patellofemoral disorders over the last two decades. Citation density, calculated as citations per year since publication, was highest among studies published between 2010 and 2015, indicating both relevance and sustained impact (Fig. 1.). This temporal distribution suggests a clustering of influential research during a period of surgical innovation and increasing clinical awareness.

### 3.2 Study designs and levels of evidence

Among the 50 most cited studies, the most common study design was case series (24 %), followed by retrospective cohort studies (20 %), with prospective cohort studies, narrative reviews and systematic reviews each comprising 12 %. Only 6 % of the articles were RCT's, highlighting a predominant reliance on observational and descriptive study designs in the most influential literature.

According to the OCEBM classification, the included articles were distributed as follows:

• Level I: 6 %
• Level II: 22 %
• Level III: 18 %
• Level IV: 26 %
• Level V: 28 %

**Fig. 2:**
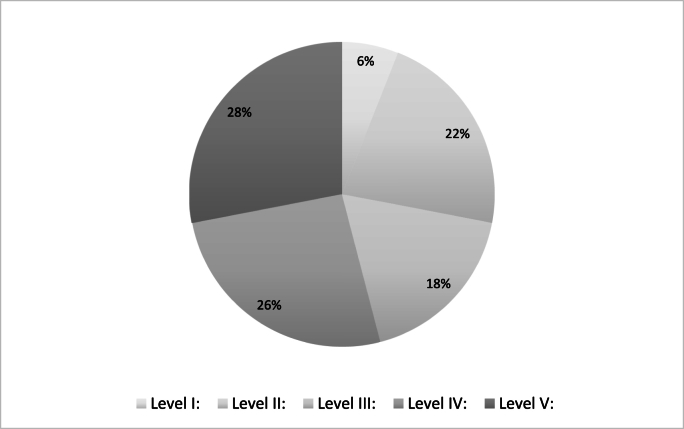
Level of evidence distribution.

This breakdown indicates the methodological limitations in the field, with over **50** **% of top-cited studies classified as Level IV or V**, reflecting a reliance on lower level evidence (Fig. 2.). While these studies have shaped current understanding and practice, they highlight the need for higher-level, prospective research to more reliably inform clinical management of patellofemoral instability.

### 3.3 Geographic and institutional distribution

**Fig. 3:**
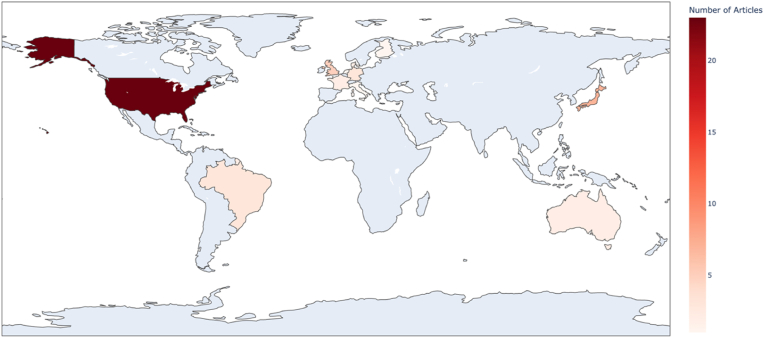
A choropleth (heat) map illustrating the number of publications originating from each country.

The 50 most cited articles originated from a geographically concentrated group of research hubs. The USA led in scholarly research output, contributing 40 % of the included studies. This was followed by France (14 %) and the UK (10 %) (Fig. 3.). Together, North America and Western Europe accounted for more than 75 % of all top-cited publications, reflecting a dominance in high-impact patellofemoral instability research from Western countries.

Other contributing countries included Germany (6 %), Japan (4 %), and Australia (2 %), while contributions from Asia, South America, and Africa were minimal or absent. No articles from institutions based in low or middle-income countries were represented among the top 50. A choropleth world map was generated to visualize the geographic distribution of the top 50 cited articles, with shading intensity reflecting the number of publications per country.

Institutionally and academically, a small number of authors contributed disproportionately to high-impact literature. **E. Nomura** emerged as the most prolific author, appearing in 5 of the top 50 cited articles, primarily focusing on MPFL reconstruction. While **Dejour's group in Lyon, France**, remains foundational, authorship trends reflect a distributed leadership across international orthopaedic centres.

Collaborative authorship across international institutions was noted in several high-impact articles, particularly those focusing on surgical outcomes and anatomical risk factor classification. However, the overall pattern indicates a concentration of authorship and influence within a relatively narrow geographic and institutional range. While geographic diversity exists, with research appearing from the USA, France, Japan, Brazil, UK etc., America and Western Europe dominate the research output.

### 3.4 Journals and publication sources

The 50 most cited articles on patellofemoral instability were published across 23 peer-reviewed journals, with a notable concentration in a few high-impact orthopaedic and sports medicine titles. The top three journals by publication count were:

• **The American Journal of Sports Medicine (AJSM)**: 9 articles (18 %)
• **Knee Surgery, Sports Traumatology, Arthroscopy (KSSTA)**: 6 articles (12 %)
• **The Knee**: 4 articles (8 %)

**Fig. 4:**
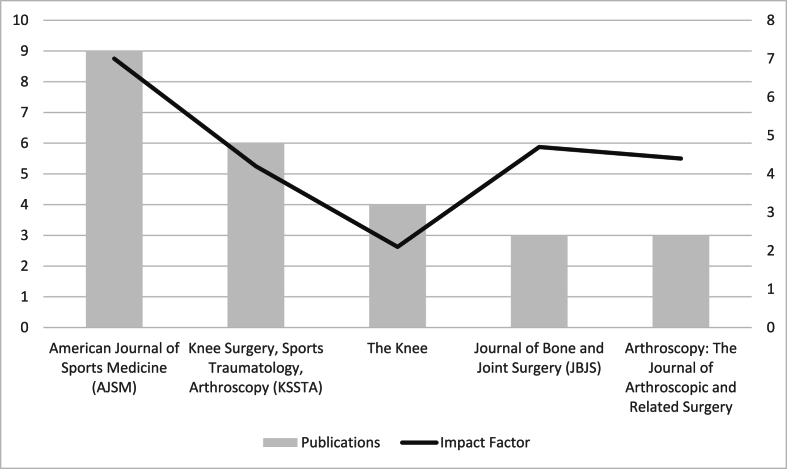
Top 5 journals and impact factor.

Together, these three journals accounted for 38 % of the top-cited articles, highlighting their key role in the distribution of impactful research in this domain (Fig. 4.).

Other prominent publication journals included:

• **The Journal of Bone and Joint Surgery (JBJS)**: 3 articles (6 %)
• **Arthroscopy: The Journal of Arthroscopic and Related Surgery**: 3 articles (6 %)

This distribution depicts the strong alignment of top-cited publications with journals that focus on surgical innovation and sports medicine. The predominance of AJSM and KSSTA reflects the surgical inclination of patellofemoral instability literature, with these journals offering both high visibility and specialized readerships in orthopaedics (Table 1.).

**Table 1:** Top 5 journals and impact factors.

Journal	Publications	Impact Factor
American Journal of Sports Medicine (AJSM)	9	7.0
Knee Surgery, Sports Traumatology, Arthroscopy (KSSTA)	6	4.2
The Knee	4	2.1
Journal of Bone and Joint Surgery (JBJS)	3	4.7
Arthroscopy: The Journal of Arthroscopic and Related Surgery	3	4.4

Conversely, journals focused on rehabilitation, biomechanics, or radiology featured only sporadically among the top-cited studies. This supports previous observations regarding the lack of representation of non-operative care and interdisciplinary perspectives in the high-impact literature, highlighting potential avenues for future research diversification.

Analysis of author contributions among the 50 most cited articles on patellofemoral instability revealed a small group of researchers with particularly high scholarly output. The most frequently publishing author was:

1. **E. Nomura – 5 publications**

Several others appeared in two publications each, including **D.C. Fithian**, **J.L.E. Gomes**, **M. Deie**, **M. Inoue**, and **SN Parikh**.

Among these, E. Nomura emerged as the most prolific contributor, appearing as a lead or co-author in 10 % of the top-cited articles. His work largely focused on MPFL reconstruction and surgical outcomes, reflecting the thematic dominance of operative strategies in the literature.

Other frequently cited authors, such as D.C. Fithian and H. Dejour, contributed foundational research on the natural history of patellar dislocation and anatomical risk stratification, respectively. **Dejour's radiographic classification of trochlear dysplasia remains a cornerstone in the diagnosis of patellofemoral instability**.^3^

### 3.5 Research focus and thematic trends

An analysis of the primary focus of each article revealed a clear emphasis on **surgical management**, which accounted for **50 % of the top 50 cited studies**. Within this category, most articles specifically addressed **MPFL reconstruction**, focusing on aspects such as graft selection, tunnel placement, fixation methods, and operative outcomes.

Beyond surgery, several additional themes were evident:

• **Imaging and diagnostics (16 %)**: Assessed patellofemoral morphology using MRI and radiographic tools, like trochlear dysplasia, TT-TG distance, and patellar tilt.
• **Biomechanical studies (16 %)**: This category included analyses of patellar tracking, graft isometry, and stress modelling using cadaveric or computational simulations.
• **Epidemiology (10 %)**: These works investigated the natural history and incidence of patellar dislocation and recurrence.
• **Complication analysis (6 %)**: Focused on adverse outcomes and failure mechanisms of surgical interventions.
• **Rehabilitation (2 %)**: Only one study addressed structured non-operative care such as physiotherapy or bracing.

This distribution highlights a pronounced bias toward surgical approaches, particularly MPFL reconstruction, while **non-operative care, return-to-sport protocols, and functional recovery pathways remain underrepresented**. This is significant given that **conservative management is often the first-line approach** for patients following a primary dislocation in the absence of osteochondral injury. ^23^

These findings point to a need for future research to fill the following gaps:

• Long-term outcomes following **non-operative management**
• Comparative effectiveness of different **rehabilitation strategies**
• Development and validation of **patient-centred outcome measures** for diverse populations

Addressing these areas will be essential for supporting **comprehensive and individualized care** in the management of patellofemoral instability.

### 3.6 Keyword Co-occurrence and network analysis

**Fig. 5:**
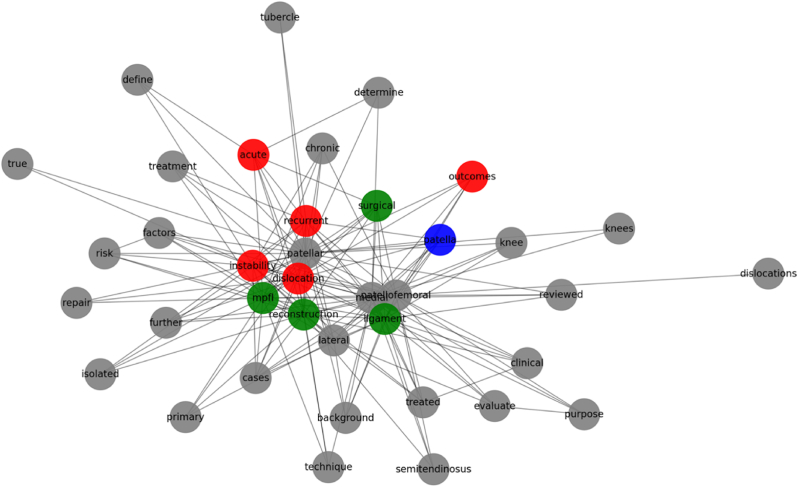
Keyword co-occurrence network of the 50 most cited articles on patellofemoral instability. Nodes represent keywords; lines indicate co-occurrence. Colours reflect thematic clusters: surgical (Green), anatomical/imaging (Blue), and outcomes/instability (Red).

A keyword co-occurrence analysis was performed using the abstracts of the top 50 most cited articles. Repeated terms were extracted and mapped into a co-occurrence network, where each **node represents a unique keyword**, and **edges indicate how often two keywords appear together** in the same abstract (Fig. 5.).

The most commonly recurring terms included:

• **“medial patellofemoral ligament (MPFL)”**
• **“reconstruction”**
• **“trochlear dysplasia”**
• **“patellar instability”**
• **“TT-TG distance”**
• **“MRI”**
• **“lateral patellar dislocation”**

Three distinct thematic clusters were identified within the network:

1. **Surgical Reconstruction Cluster (Green)**: The most dominant cluster, featuring keywords related to MPFL reconstruction, graft choice, surgical fixation, and clinical outcomes. It underscores the literature's strong emphasis on operative management techniques.
2. **Anatomical Risk Assessment Cluster (Blue)**: This cluster encompassed terms related to imaging modalities and structural risk factors, including patella alta, trochlear dysplasia, and TT-TG distance.
3. **Outcome and Instability Cluster (Red)**: A smaller cluster centred on terms such as “recurrent,” “acute,” “dislocation,” and “outcomes,” highlighting research concerned with recurrence rates and clinical prognosis following patellar dislocation.

Collectively, these findings depict a field shaped by **technical innovations in surgery and radiographic evaluation**, yet with notable gaps in **conservative management strategies**, **long-term functional outcomes**, and **patient-centred care**. Future research that addresses these gaps will be critical in supporting a more comprehensive, evidence-based approach to patellofemoral instability.

## 4 Discussion

This bibliometric analysis provides a comprehensive overview of the most influential literature on patellofemoral instability, revealing the dominant themes, methodological patterns, and areas of underrepresentation within the field. The most highly cited publications are largely concentrated within a small number of journals and academic institutions and focus primarily on surgical treatment, especially MPFL reconstruction. Despite the high citation counts, most of these studies offer low levels of evidence, with the majority classified as Level IV or V.

The central emphasis on surgical interventions, particularly MPFL reconstruction, aligns with current clinical trends and reflects efforts to refine operative techniques, graft selection, and radiographic parameters such as trochlear dysplasia and TT–TG distance. ^3^^,^^6^^,^^13^ These topics were the most frequently appearing keywords in our co-occurrence analysis, highlighting their foundational role in the literature. However, while this surgical focus has yielded advances in technique and anatomical classification, it has led to the relative neglect of non-operative care and rehabilitation strategies in high-impact research.

This imbalance is particularly notable given that conservative management remains the first-line treatment for many patients following a first-time dislocation, especially in the absence of osteochondral injury. ^2^^,^^4^^,^^5^^,^^7^ Structured rehabilitation, physical therapy, and return-to-sport protocols are critically important to long-term patient function, yet they are markedly underrepresented in the most cited articles. Furthermore, few studies integrate PROMs, quality of life indices, or functional recovery benchmarks, elements that are increasingly central in other areas of orthopaedic research, such as rotator cuff pathology and total hip arthroplasty. ^20^^,^^21^

Authorship and geographic trends reveal a concentration of influence among a small group of contributors. E. Nomura was the most prolific author, contributing to 10 % of the top-cited articles, while the group led by Dejour in Lyon, France, continues to have a foundational impact through their work on trochlear dysplasia classification. ^3^ North America and Western Europe accounted for the majority of publications, with the USA contributing 40 % of the total, highlighting a significant geographic skew in research output. This concentration may limit the generalizability of findings to diverse healthcare settings, particularly in low and middle-income regions where diagnostic and surgical resources may differ substantially.

In the context of keyword co-occurrence analysis, three dominant thematic clusters emerged: surgical reconstruction, anatomical risk assessment, and clinical outcomes. Terms related to rehabilitation, conservative care, or return-to-sport guidance were rarely evident. This further confirms a prevailing research focus on anatomical correction and operative management, often at the expense of broader, patient-centred considerations.

As the field moves forward, future research should prioritize closing these gaps. There is a need for more prospective, high-level evidence, increased inclusion of varying geographic regions, and stronger emphasis on conservative management, functional outcomes, and quality-of-life measures. ^10^ Longitudinal studies evaluating long-term outcomes, including recurrence rates and osteoarthritis development, are also essential. By broadening research priorities to reflect a more comprehensive view of patient care, the field of patellofemoral instability can better support evidence-based, individualized treatment approaches.

### 4.1 Limitations

This study has several limitations inherent to bibliometric analyses. The use of Google Scholar via the Publish or Perish software offers comprehensive coverage but may include lack the indexing precision of curated databases such as Web of Science or Scopus. Citation counts, while indicative of influence, do not necessarily reflect methodological quality or clinical relevance and can be affected by factors such as article age, journal impact factor, and self-citation practices.

Additionally, this analysis was limited to the 50 most cited articles, which may have excluded recent, innovative, or niche studies that have yet to accumulate substantial citations. The categorization of study design, thematic focus, and level of evidence involved some degree of subjective interpretation by reviewers, potentially introducing classification bias despite efforts to apply standardized criteria.

## 5 Conclusion

This bibliometric analysis provides a comprehensive overview of the most influential literature on patellofemoral instability, revealing a research landscape predominantly centred on surgical reconstruction, particularly MPFL procedures. While these studies have advanced the understanding and management of patellofemoral instability, the current evidence base is largely composed of lower-level studies, with a notable lack of high-quality, prospective clinical trials.

Moreover, conservative treatment strategies, rehabilitation protocols, and patient-centred outcome measures remain significantly underrepresented in the most cited literature, representing key gaps that deserve targeted research attention.

To move the field forward, future investigations should prioritize methodological rigor, diversify participant populations and study settings, and address the full continuum of care—from diagnosis and acute management to long-term functional recovery. A more balanced and comprehensive research agenda will support the development of evidence-based, patient-focused care strategies that can improve outcomes for individuals affected by patellofemoral instability.

## CRediT authorship contribution statement

**Alexander Price:** Conceptualization, Data curation, Formal analysis, Manuscript Composition. **Nicolaas Kotze:** Formal analysis, Manuscript Composition. **Xander van Heerden:** Formal analysis, Manuscript Composition. **Gerard A. Sheridan:** Manuscript Review, Supervision.

## Guardian/patient consent

Not Applicable.

## Ethics

This study relied exclusively on publicly accessible bibliometric data and did not involve human participants or personal health information. As such, it was exempt from institutional review board approval and adhered to ethical standards governing secondary research.

## Funding

This research did not receive any specific grant from funding agencies in the public, commercial, or not-for-profit sectors.

## Declaration of interest

None.
